# Association mapping for general combining ability with yield, plant height and ear height using F_1_ population in maize

**DOI:** 10.1371/journal.pone.0258327

**Published:** 2021-10-15

**Authors:** Yunxiao Zheng, Xintong Han, Yongfeng Zhao, Liying Zhu, Yaqun Huang, Xiaoyan Jia, Zhongqin Zhang, Jingtang Chen, Jinjie Guo

**Affiliations:** 1 Hebei Sub-Center of National Maize Improvement Center, Key Laboratory Jointly Constructed by the Ministry of Education and Hebei Province, Key Laboratory for Crop Germplasm Resources of Hebei, North China Key Laboratory for Crop Germplasm Resources under the Ministry of Education, College of Agronomy, Hebei Agricultural University, Baoding, China; 2 College of Agronomy, Qingdao Agricultural University, Qingdao, China; KGUT: Graduate University of Advanced Technology, ISLAMIC REPUBLIC OF IRAN

## Abstract

General combining ability (GCA) is an important index for inbred lines breeding of maize. To identify the genetic loci of GCA and associated agronomic traits, an association analysis with 195 SSRs was made in phenotypic traits of 240 F_1_ derived from 120 elite inbred lines containing current breeding resources of maize crossed with 2 testers (Zheng58 and Chang7-2) in two places in 2018. All of the 20 association loci detected for grain yield (GY), plant height (PH), ear height (EH) and GCA for the three traits in two places could explain a phenotypic variation range of 7.31%-9.29%. Among the 20 association loci, 9 (7.31%-9.04%) were associated with GY, 4 (7.22%-8.91%) were related to GCA of GY, 1 (7.56%) was associated with PH, and 3 (7.53%-8.96%) were related to EH. In addition, 3 loci (9.14%-9.29%) were associated with GCA of PH whereas no locus was identified for GCA of EH. In the comparison of the association loci detected in Baoding and Handan, interestingly, one locus (7.69% and 8.11%) was identified in both environments and one locus (7.52% and 7.82%) was identified for yield and GCA of yield. Therefore, the identification of GY-, PH-, EH- and GCA-related association loci could not only provide references for high yield breeding of maize, but also help us comprehend the relationships among GY, agricultural traits and GCA.

## Introduction

Maize (*Zea mays* L.) is one of the main food crops, feeds and industrial raw materials, as well as one of the most cultivated crops worldwide [1]. Therefore, the high and stable yield has been a primary long-term target for maize breeding. As a complex quantitative trait controlled by multigene [2, 3], yield had a low heritability and was easily influenced by extragenetic factors, however the composition factors of yield had high stability and heritability [4]. Robinson et al. [5] found that choosing the traits related to yield was more effective than choosing yield trait straight in breeding modification. According to Beavis [6], plant height (PH) had a close relationship with grain yield (GY) and was also related to ear height (EH) [7]. Therefore, a reasonable PH and EH are beneficial for increasing maize yield.

To date, lots of quantitative trait loci (QTL) and candidate genes related to maize GY, PH and EH have been identified. He et al. [8] identified 10 QTLs for PH in 150 recombinant inbred line (RIL) populations under six environments. In different environments, for PH and EH, 41 SNPs were detected and 19 candidate genes were identified [1]. In addition, several genes related to GY, PH and EH have been cloned and identified, such as *d8*, *d9*, *ZmGA3ox2*, *TD1* and *FEA2* [9–12]. However, the genetic architecture of GY, PH and EH remains unclear in the F_1_ population. Dissecting the genetic basis of GY, PH and EH would provide a theoretical direction for maize breeding.

Heterosis has been extensively exploited in the process of maize breeding and genetic factors contributing to heterosis is combining ability (CA) of parents. Thus, inbred lines are prerequisite for hybrid development in maize. CA, defined as general combining ability (GCA) and specific combining ability (SCA), is an important parameter in evaluating inbred lines. Since Sprague and Tatum [13] put forward the concepts of GCA and SCA, it has been a major strategy to increase grain yield (GY) to apply CA to crop breeding, especially maize breeding, with a series of genetic mating designs. GCA is the average performance of F_1_ traits. Compared with SCA for specific combinations, using GCA to select inbred lines and elite combiners as the guideline is more accurate and efficacious. Abdelmoneam et al. [14] found that GCA was more vital than SCA inheritance of yield. Increasing yield which is the ultimate object of maize breeding is confronted with enormous challenges. Analyzing GCA for the yield of F_1_ deriving from inbred lines would be a profitable method for high yield breeding. Pswarayi and Vivek [15] found one potential tester by analyzing CA among early maturing maize. Abdelmoneam et al. [14] found some specific mating combinations were useful for breeding modification of maize and Abdel-Mone et al. [16] indicated that mean squares of hybrid’s combining ability of yield trait, 100-kernel weight, and other four agronomic traits, except ear diameter, were highly significant. Kirjavainen et al. [17] found that GCA effect had significant mean squares for 1000-kernel weight and plant height.

Simple sequence repeat (SSR), also known as microsatellites, is broadly used because they are codominant, highly polymorphic, multi-allelic and have a high polymorphism information content [18]. As an effective method, association mapping has higher mapping resolution relative to linkage mapping. In previous study, SSR markers have played an important role in assessing genetic diversity and population structure among crop germplasm resources [19–21]. In addition, loci related to phenotypic traits were detected in maize, such as seed storability [22], grain yield [23], and maize starch content [24].

In this study, an association analysis was made for GY, PH, EH and GCA of the three studied traits with 195 SSRs in F_1_. Association loci detected in parental lines might be in the place hided in F_1_ owing to allelic interaction. On this count, using F_1_ to make the association analysis would achieve more accurate results. The objectives of the current study were to (1) determine GCA of 120 inbred lines and seek out the tested lines with high GCA; (2) detect loci associated with GY, PH, EH and GCA of the studied traits.

## Material and methods

### Plant materials

A total of 240 F_1_ hybrids derived from 120 maize inbred lines that were crossed with 2 elite inbred lines—Chang7-2 and Zheng58—were employed in this study. The 120 maize inbred lines included a core collection of maize germplasm resources of China, derived lines and lines selected from American hybrid. Among them, a portion of core collection resources covered the current resource background of maize breeding basically and had abundant genetic variation. In addition, these inbred lines cover the heterosis groups of core inbred lines in China. Chang7-2 and Zheng58 belonged to Tang SPT and Reid population, respectively, and they were parents of Zhengdan958 which had the largest planting area of 4.0×10^6^ hectares with 20% production of maize [25]. 120 association analysis inbred lines and 2 test inbred lines were crossed in NCⅡdesign at Hainan maize breeding experiment station in the winter of 2017. For all the 240 F_1_ crosses, a randomized block design with two replications was used in this experiment. Each material was planted in a plot with two rows in 0.60m inter-row spacing, 4.0m long row and 0.25 m plant to plant spacing using a population density of 65,000 plants per hectare at the Experimental Station of Hebei Agricultural University in Baoding (115.48°E, 38.85°N) and Experimental Station Agricultural Academy of Handan (114.47°E, 36.60°N) in the summer of 2018. All the plants, around with guarding rows, were in field management using local maize tillage methods throughout the growth periods. Drought stress did not occur during the growing season in either year.

### Phenotype data collection

PH (cm) and EH (cm) of 10 plants from the third to the seventh plant every row were measured continuously in each plot after maize anthesis and silking. Base of plant to the top of the first tassel branch and node bearing of the primary ear were taken as the distance of PH and EH [26]. All ears, except the first and the last ears of each line, were harvested after the mature stage of the maize materials. Ten typical ears were selected after they were dried naturally with a moisture content of below 18% for the purpose of analyzing yield traits.

### Phenotype analysis

Phenotype data (GY, PH and EH) were dealt and calculated GCA following the method developed by Wen et al. [27]. Descriptive statistics and analysis of the combining ability effect were carried out using SPSS statistics v21.0 software. The statistical model to calculate GCA was $g_{i}=\bar{y_{i.}}-\bar{y_{..}}$ where *g_i_* is the GCA effect of inbred lines i; $\bar{y_{i.}}$ is the average value of inbred line i and two testers. $\bar{y_{..}}$ is the average value of all mating designs.

### SSR genotyping

The seedlings of 120 inbred lines and 2 test lines were cultured in the greenhouse, and the DNA data of seedlings were extracted using CTAB method at four leaf stages of the test population and diluted to 50 ng/ μL. The purity and concentration of DNA was assessed by optical density (OD260/OD280) and the quality of DNA was detected by 0.8% agarose gel electrophoresis. 432 SSRs primer pairs obtained from Maize GDB (**http://www.maizegdb.org/**), which ensured even distribution of all the SSRs on chromosome. Then 195 SSRs primer pairs with high polymorphism and a clear band were got using 10 inbred lines that were selected randomly as samples from 120 inbred lines. All of the 195 SSRs primer pairs were distributed on the whole genome of maize. Genotype of 120 inbred lines and 2 test lines were identified via PCR amplification and electrophoresis using the method offered by Xu et al. [28]. The SSR amplification reactions were carried out in 96-well microtiter plates using a Bio-Rad Thermal Cycler T100 (USA). Differential banding patterns were recorded using 0, 1, 9, respectively, for no band, having a bright band and missing band in the same mobility position. All records were used to build a genotype database.

### Population structure analysis

A set of 195 SSRs distributed on 10 chromosomes were selected to identify the structure of 120 inbred lines using Structure v2.3.4 software [29]. The Structure software with a bayesian clustering method performed three runs for K (set from 2 to 10) to calculate the genetic components. Meanwhile, a parameter of 500,000 were set both to MCMC and length of burn-in period in each run. According to the maximum likelihood criterion, one most suitable K value was determined with genealogy of inbred lines using the method performed by Evanno [30] and following the model below

Δk=m|L(K+1−2L(K)+L(K)|/s|L(K)|)

Genetic component (Q value) was employed 50% as the dividing line to determine which population the inbred lines belonged to. Lines with probabilities of membership greater than 50% were placed into the related groups, while those with membership probabilities lower than 50% were allotted to a “mixed” group [31].

### Genetic diversity analysis

A database of the genotype data of 120 inbred lines was used to calculate the gene diversity index, the number of alleles per locus, and the polymorphism information content (PIC) which was the most extensive parameter to evaluate genetic diversity using Power Marker v3.25 [32] following the statistical models below

$$H=(\frac{n}{n-1})(1-\sum pij^{2})$$

$$PIC_{i}=1-\sum p_{i}{}^{2}-\sum \sum 2p_{i}{}^{2}p_{j}{}^{2}$$

Where *H* is the genic diversity of certain locus; *n* is the amount of materials; *p_ij_* is the frequency of alleles variation of the i^th^ site and the j^th^ site alleles; *p_i_* and *p_j_* are the frequency of the i^th^ and the j^th^ alleles.

### Linkage Disequilibrium (LD)

LD analysis of 240 F_**1**_ was evaluated with parameters r^**2**^ (squared allele frequency correlations) and D’ (Linkage disequilibrium coefficient) for SSR marker pairs using TASSEL V3.0 with sliding window size at 50. Allele loci was regarded as linkage disequilibrium when P<0.01.

### Association analysis

Genome-wide association study was evaluated using TASSEL V3.0 program [33] based on mixed linear mode (MLM). 195 SSRs overlapping wide genome and 3 trait phenotypes together with population structure matrix (Q matrix) and Kinship matrix (K matrix) which acted as covariate to decrease spurious association [34] were calculated to detect marker loci combining with target traits.

## Results

### Phenotype analysis

Trait phenotypes and combining ability were analyzed in this study. Results were shown in Table 1. Trait phenotype showed that PH of F_**1**_ ranged from 172.80cm to 267.80cm with the tester of Zheng58 for the materials of and 185.90cm to 322.80cm with Chang7-2 in addition, EH of F_**1**_ ranged from 64.60cm to121.20cm with Zheng58 and 92.60cm to 165.60cm with Chang7-2. As described in this table, the same trait of one tester had a similar drift but a significant difference. Furthermore, the differences of the traits between Baoding and Handan indicated that GY, PH and EH might be susceptible to environment which meant environment variables should be taken seriously in breeding programs. In addition, GCA of yield ranged from -0.63 to 0.65 in Baoding and from -0.79 to 1.11 in Handan. GCA of PH and EH ranged from -31.38 to 62.77 and from -18.41 to 33.49. Moreover both the highest GCA were resulted by materials 78599. The result of analysis of variance for GY was listed in Table 2. As shown as Table 2, the effects of environment and genotype were significant at 0.01 level, indicating the important roles of both genotypes and environment.

**Table 1:** Statistic summary of yield-related traits and GCA.

Trait	Environment	Tester	Minimum	Maximum	Average	Standard	Skewness	Kurtosis
			value	value		Deviation		
GY	Baoding	Z58	1.57	2.89	2.22	0.31	0.13	-0.83
		C7-2	1.35	3.31	2.39	0.32	-0.21	0.53
	Handan	Z58	1.63	3.67	2.59	0.42	0.10	-0.19
		C7-2	1.36	3.78	2.35	0.51	0.37	-0.50
PH	Baoding	Z58	172.80	267.80	212.67	17.27	0.54	1.18
		C7-2	185.90	322.80	242.33	24.56	0.49	0.67
EH	Baoding	Z58	64.60	121.20	90.09	11.20	0.27	0.04
		C7-2	92.60	165.60	119.56	15.04	0.72	0.40
GY GCA	Baoding		-0.63	0.65	0.00	0.25	0.02	-0.06
	Handan		-0.79	1.11	0.00	0.38	0.21	-0.30
PH GCA	Baoding		-31.38	62.77	-0.12	17.59	0.73	0.96
EH GCA	Baoding		-18.41	33.49	-0.10	10.74	0.65	0.29

**Table 2:** Analysis of variance for GY.

Trait	F-value				
	Block	Environment	Genotype	Environment × Genotype	Block × Genotype
GY	0.956	23.153**	1.823**	0.923	0.863

**: Significant difference at 0.01 probability level.

### Population structure analysis

**Fig 1:**
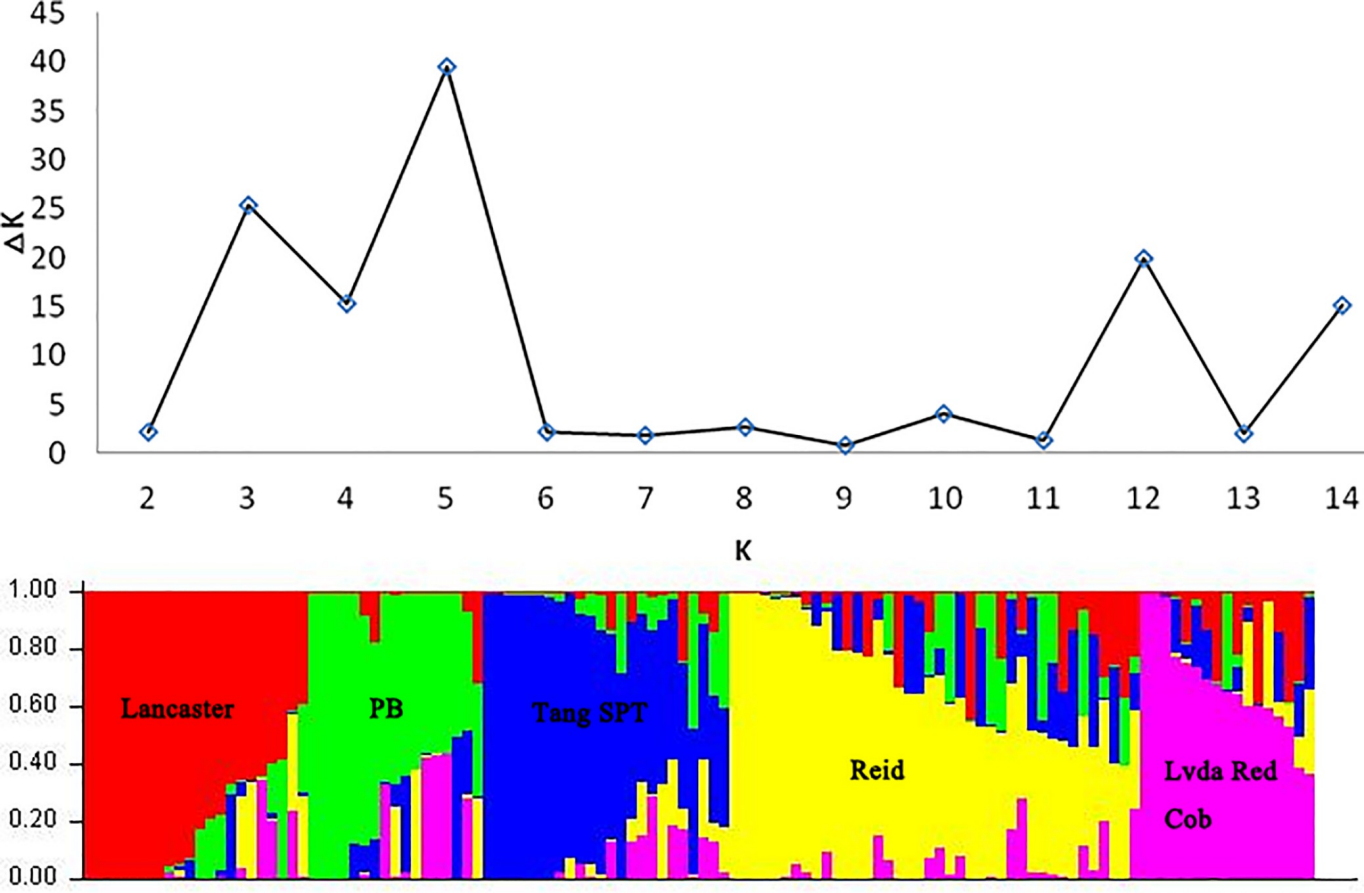
Population structure of 120 maize inbred lines.

Results of population structure analysis were shown in Tables 3 and 4. K value and ΔK were evaluated (Fig 1A). The peak value of ΔK was observed when K = 5. Therefore, an ideal structure of the research population was divided into 5 subpopulations, namely Lancaster, PB, Tang SPT, Reid and Lvda Red Cob (LRC) (Table 4, Fig 1B). Accordingly, the structure of the research population was not complex and was satisfactory for applying the population structure analysis.

**Table 3:** Q-values of 120 inbred lines.

Number	Inbred lines	Reid	Lancaster	PB	LRC	Tang SPT
1	18	0.002	0.002	0.989	0.004	0.002
2	196	0.002	0.003	0.002	0.446	0.547
3	802	0.026	0.009	0.547	0.408	0.009
4	953	0.224	0.003	0.116	0.652	0.005
5	1614	0.746	0.009	0.225	0.016	0.004
6	7236	0.233	0.145	0.385	0.229	0.008
7	7884	0.023	0.862	0.010	0.095	0.010
8	7922	0.988	0.003	0.004	0.003	0.002
9	8982	0.103	0.379	0.395	0.118	0.004
10	9058	0.398	0.003	0.188	0.378	0.033
11	9710	0.638	0.319	0.020	0.020	0.003
12	20564	0.031	0.115	0.149	0.555	0.150
13	2005–4	0.123	0.428	0.234	0.137	0.078
14	68122	0.004	0.053	0.937	0.003	0.003
15	68139	0.008	0.174	0.763	0.053	0.003
16	78599	0.007	0.008	0.588	0.394	0.003
17	1205A	0.671	0.306	0.005	0.015	0.003
18	18–599	0.013	0.003	0.963	0.013	0.008
19	200B	0.011	0.194	0.002	0.768	0.024
20	3489a	0.019	0.127	0.019	0.700	0.135
21	3H2	0.011	0.182	0.097	0.319	0.391
22	433–7	0.312	0.434	0.036	0.216	0.002
23	468–3	0.534	0.433	0.005	0.013	0.015
24	698–3	0.317	0.004	0.010	0.667	0.003
25	7026B	0.009	0.007	0.062	0.021	0.901
26	7903E	0.746	0.057	0.008	0.043	0.145
27	806A	0.345	0.535	0.111	0.007	0.003
28	807D	0.464	0.015	0.264	0.252	0.005
29	811A	0.003	0.009	0.852	0.020	0.116
30	A679	0.881	0.003	0.004	0.021	0.092
31	B73	0.643	0.313	0.017	0.022	0.004
32	B98	0.004	0.639	0.005	0.290	0.062
33	BM	0.007	0.639	0.005	0.324	0.024
34	C521	0.240	0.005	0.673	0.077	0.005
35	Chang3	0.201	0.462	0.004	0.312	0.020
36	Chang72	0.032	0.225	0.028	0.373	0.342
37	CN104	0.216	0.407	0.029	0.305	0.043
38	D1051	0.053	0.005	0.935	0.004	0.003
39	D1139	0.318	0.009	0.638	0.008	0.026
40	d140	0.282	0.057	0.399	0.250	0.012
41	D20	0.169	0.117	0.704	0.007	0.003
42	D33A	0.021	0.173	0.005	0.021	0.779
43	D88	0.006	0.179	0.738	0.074	0.003
44	Dan 9046	0.037	0.225	0.173	0.555	0.009
45	Dan340	0.184	0.034	0.028	0.751	0.003
46	DF24	0.988	0.005	0.002	0.002	0.002
47	DF32	0.006	0.977	0.008	0.005	0.004
48	DH138	0.038	0.026	0.924	0.003	0.009
49	DM07	0.324	0.664	0.005	0.004	0.003
50	e qun 3	0.368	0.013	0.418	0.120	0.080
51	E200	0.003	0.005	0.385	0.573	0.034
52	E588	0.324	0.185	0.003	0.144	0.344
53	E600	0.235	0.035	0.720	0.007	0.004
54	E601	0.296	0.103	0.140	0.458	0.003
55	FAP1360A	0.103	0.635	0.017	0.044	0.201
56	H21	0.424	0.006	0.002	0.375	0.192
57	Hai9-21	0.012	0.008	0.298	0.672	0.010
58	Huangchang a	0.014	0.100	0.178	0.013	0.695
59	Huangchang b	0.004	0.017	0.539	0.226	0.214
60	Huangzao3	0.007	0.010	0.002	0.040	0.940
61	Ji444	0.002	0.005	0.008	0.353	0.632
62	Ji63	0.033	0.582	0.039	0.009	0.337
63	KP3130	0.229	0.734	0.014	0.012	0.010
64	L061F	0.746	0.032	0.010	0.011	0.201
65	L-1	0.003	0.005	0.985	0.002	0.005
66	L473	0.049	0.303	0.077	0.529	0.041
67	La2-4	0.404	0.407	0.008	0.041	0.140
68	LM-2	0.157	0.155	0.659	0.018	0.011
69	Lv28	0.041	0.314	0.174	0.467	0.003
70	M101	0.036	0.294	0.367	0.003	0.301
71	M1016	0.221	0.044	0.241	0.476	0.017
72	M14	0.350	0.453	0.005	0.188	0.005
73	M22	0.557	0.096	0.004	0.341	0.003
74	Max	0.437	0.545	0.005	0.009	0.004
75	Mo17	0.220	0.757	0.007	0.006	0.010
76	N68a	0.205	0.006	0.382	0.011	0.396
77	Nan21-3	0.395	0.496	0.003	0.014	0.092
78	Niu2-1	0.008	0.161	0.003	0.434	0.394
79	P007	0.033	0.339	0.216	0.378	0.035
80	P167	0.008	0.557	0.361	0.061	0.014
81	PH4CV	0.144	0.150	0.134	0.544	0.027
82	PH6WC	0.746	0.229	0.003	0.020	0.002
83	Q1261	0.423	0.113	0.085	0.062	0.317
84	Qi205	0.017	0.438	0.059	0.334	0.152
85	Qiong51	0.367	0.008	0.007	0.613	0.005
86	R08	0.462	0.334	0.117	0.063	0.023
87	R150	0.003	0.002	0.990	0.003	0.002
88	R1656	0.009	0.011	0.184	0.502	0.294
89	R31	0.061	0.038	0.233	0.662	0.005
90	SC14	0.004	0.011	0.254	0.706	0.025
91	SC24-1	0.414	0.005	0.220	0.352	0.010
92	SC30-1	0.231	0.006	0.411	0.345	0.007
93	Shan89	0.005	0.006	0.983	0.003	0.002
94	Shen137	0.002	0.002	0.993	0.002	0.001
95	Shen977	0.002	0.003	0.992	0.002	0.002
96	Song1145	0.002	0.002	0.992	0.002	0.002
97	Su75	0.894	0.061	0.005	0.012	0.028
98	Suwan1611	0.075	0.330	0.009	0.342	0.244
99	T24	0.554	0.309	0.007	0.121	0.008
100	W172	0.611	0.062	0.004	0.320	0.003
101	W222	0.872	0.006	0.102	0.012	0.008
102	W238	0.789	0.009	0.025	0.170	0.008
103	W344	0.786	0.159	0.017	0.022	0.016
104	w499	0.765	0.017	0.124	0.020	0.074
105	W668	0.763	0.033	0.195	0.005	0.004
106	W967	0.580	0.008	0.034	0.085	0.293
107	W968	0.583	0.006	0.390	0.009	0.013
108	XF223	0.026	0.165	0.237	0.569	0.003
109	XOP2	0.567	0.415	0.004	0.010	0.003
110	Xun971	0.286	0.018	0.286	0.011	0.398
111	Y223	0.008	0.482	0.113	0.378	0.018
112	y9961	0.063	0.235	0.574	0.095	0.033
113	Ye478	0.988	0.003	0.003	0.003	0.003
114	Ye52106	0.029	0.325	0.004	0.628	0.014
115	Ye8001	0.990	0.003	0.002	0.002	0.003
116	Ye8112	0.883	0.016	0.081	0.014	0.005
117	Ye832	0.990	0.003	0.004	0.002	0.002
118	Zheng32	0.946	0.024	0.010	0.012	0.007
119	Zi330	0.989	0.003	0.004	0.002	0.002
120	Zong3	0.967	0.006	0.012	0.013	0.003

**Table 4:** Population division of 120 inbred lines.

Group	Inbred lines
Reid	Ye478, Zi330, 7922, Ye8112, L061F, PH6WC, 1205A, DF24, w499, XOP2, 9710, 468–3, Zheng32, T24, Su75, 7903E, W172, W238, W668, 1614, W344, Ye832, W968, M22, A679, W222, Zong3, W967, Ye8001, B73
Lancaster	806A, Max, P167, 7884, FAP1360A, DM07, B98, KP3130, BM, PH4CV, Ji63, DF32, Mo17
Tang SPT	D33A, 7026B, Ji444, Huangzao3, 196, Huangchang a
Lvda Red Cob	Dan340, 200B, Qiong51, Ye52106, 3489a, XF223, 953, R1656, R31, L473, Dan 9046, 698–3, E200, SC14, 20564, Hai9-21
PB	Shen137, 68122, D88, y9961, 78599, 802, Song1145, E600, D1139, C521, 68139, D20, Shen977, Huangchang b, 18–599, DH138, LM-2, L-1, 18, 811A, R150, Shan89
Mixed population	Lv28, 9058, Nan21-3, R08, 2005–4, 7236, 807D, Y223, Q1261, 433–7, P007, CN104, Niu2-1, Suwan1611, SC30-1, N68a, 8982, La2-4, e population 3, Chang72, D1051, 3H2, M101^6^, H21, SC24-1, M14, M101, d140, E601, E588, Chang3, Qi205, Xun971

### Kinship analysis

**Fig 2:**
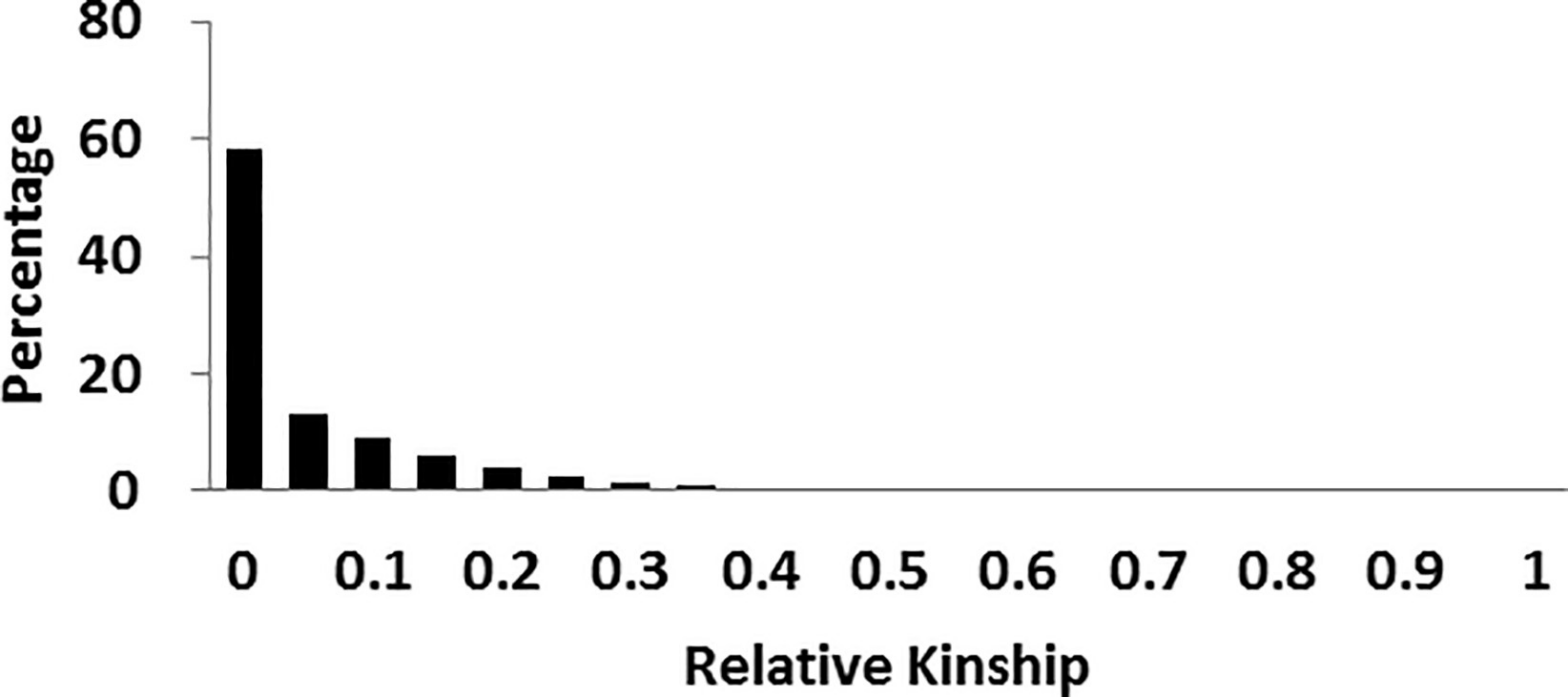
Distribution of pairwise relative kinship among 120 inbred lines.

Kinship analysis of the 120 inbred lines was carried out in combination with 195 SSR markers using SPAGeDi-1.3d software. Results showed that 58% of the population had no relationship for relative kinship value (K) equaling to 0 and only 6% of the materials had high kinship with K>0.5 (Fig 2).

### Genetic diversity analysis

**Fig 3:**
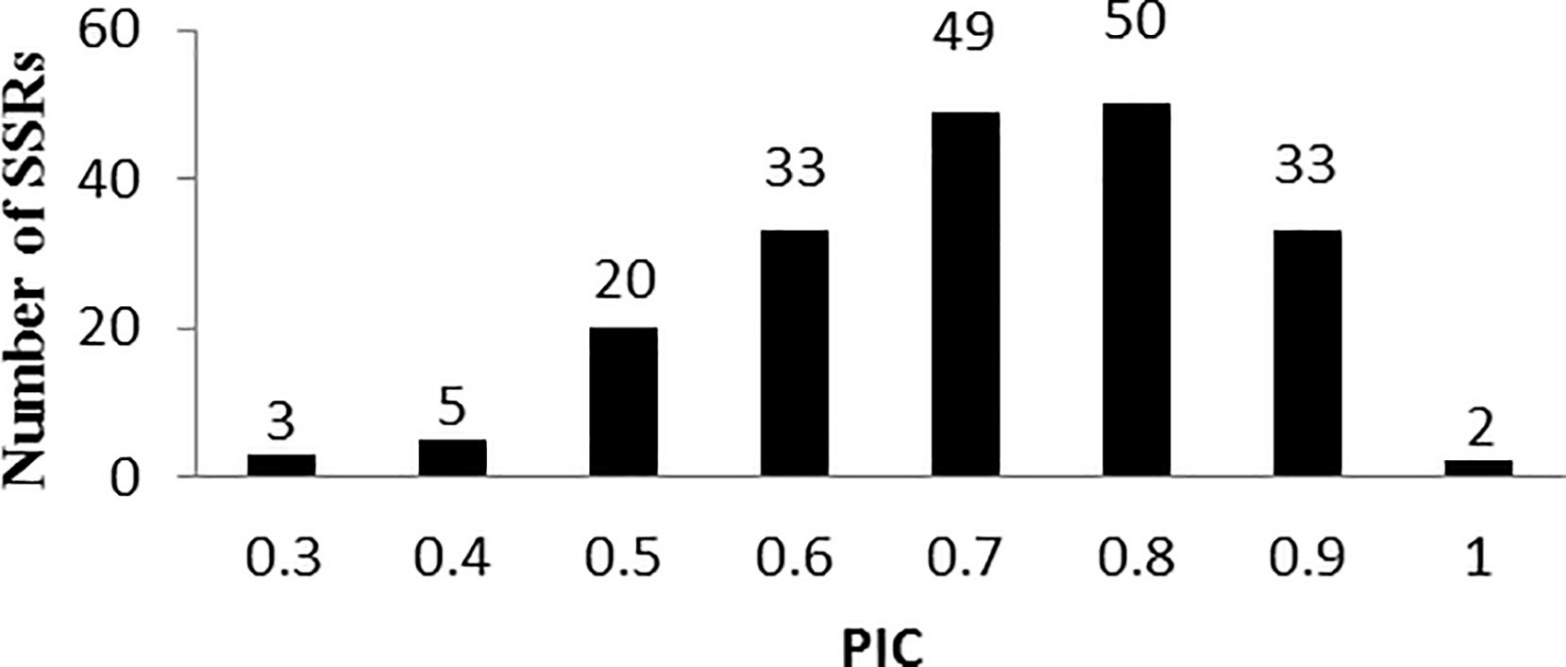
Histogram of PIC distribution of 195 SSRs.

Genetic diversity was analyzed within 120 maize inbred lines in combination with 195 SSRs which covered genome wide using Powermarker v3.25 software. 1,478 allelic variations were obtained and 2~26 loci were detected by one marker with an average of 7.58 loci. In this experiment, the genetic diversity ranged from 0.2950 (umc1794) to 0.9235 (bnlg1863) with an average value of 0.7016, and from 0.2803 (umc1794) to 0.9182 (bnlg1863) for polymorphic information content (PIC) with an average value of 0.6593. The frequency distribution is shown in Fig 3. The largest proportion among the 195 SSRs was the markers with PIC value of 0.7 and 0.8 accounting for 50.7%. And 85.6% of the markers was in PIC value over 0.6. Compared with the result of Li [35] (PIC = 0.6), an approximate result was shown in this experiment which meant the research population had a desirable genetic diversity and was suitable to association analysis.

### Linkage Disequilibrium (LD) analysis

LD, the foundation of association analysis of 120 inbred lines association analysis, was detected using TASSEL V3.0 software. Results of LD pairwise detection showed that 5.21% of the pairwise markers were significant when P<0.01, and 12.38% of the pairwise markers were in LD when D’>0.5. D’ and r^**2**^ had an average level of 0.480 and 0.041 of the pairwise markers, respectively. In addition, D’ and r^**2**^ had an average of 0.638 and 0.214 for significant pairwise markers, respectively.

### Marker loci associated with GY, PH and EH

An association analysis within 195 SSRs and GY, PH and EH was made in our research.–log_**10**_^**p-value**^ was taken as the measuring parameter and the marked loci and trait phenotypes were considered as significant association if–log_**10**_^**p-value**^ >2.5,.

When–log_**10**_^**p-value**^ >2.5, a total of 13 significant associating marker loci were detected in both Baoding and Handan (Table 5) among which 9 loci were associated with yield trait and 1 was associated with PH, besides 3 loci were associated with EH. Furthermore only one locus umc1794 distributed on chromosome 9 (bin9.04) was detected in Baoding contributed by tester Chang7-2 and explained 7.31% of the phenotypic variation. 3 association loci (mmc0282, mmc0241, umc1061) contributed by Zheng58 were detected mapped on bin 5.05, 6.06 and 10.07 explained 7.97%, 7.69% and 7.52% of the phenotypic variation, respectively.

**Table 5:** Marker loci associated with GY, PH, EH.

Trait	Environment	Tester	Locus	Bin	Genetic distance	-log_10_(P)	R^2^(%)
F_1_ yield/Kg	Baoding	C7-2	umc1794	9.05	427.61	2.55	7.31
		Z58	mmc0282	5.05	396.46	2.70	7.97
			mmc0241	6.05	312.78	2.62	7.69
			umc1061	10.06	392.52	2.58	7.52
	Handan	C7-2	Fdx2	6.00	9.10	2.82	8.32
			mmc0241	6.05	312.78	2.84	8.11
			umc2393	9.00	21.30	3.10	9.04
			bnlg430	9.03	242.02	2.75	7.80
			umc2093	9.01	85.58	2.63	7.37
PH	Baoding	Z58	umc1178	6.02	145.30	2.66	7.56
EH	Baoding	C7-2	umc2386	5.05	428.00	2.91	8.96
			umc1710	7.04	410.50	2.71	8.18
			umc1366	9.05	489.90	2.53	7.53

In Handan, a total of 5 significant association marker loci were detected. Fdx2 (bin6.00) the only one locus contributed by tester Chang7-2 was mapped on chromosome 6 and explained 8.32% of the phenotypic variation. 4 loci mmc0241 (bin 6.05), umc2393 (bin9.00), bnlg430 (bin 9.03), umc2093 (bin9.01) of the five were contributed by Zheng58, distributed on chromosome 6, 9 and explained 8.11%, 9.04%, 7.80% and 7.37% of the phenotypic variation, respectively.

For PH, 1 locus (umc1178/bin6.02) mapped on chromosome 6 was detected in Zheng58 test population explained 7.56% of the phenotypic variation in Baoding. 3 loci umc2386 (bin5.05), umc1710 (bin7.04) and umc1366 (bin9.05) associated with EH were detected in Chang7-2 test population distributed on chromosome 5, 7and 9 explained 8.96%, 8.18% and 7.53% of the phenotypic variation, respectively.

### Marker loci associated with GCA of GY, PH and EH

A total of 7 association loci were detected for GCA (Table 6). 3 loci associated with Yield GCA of the 7 were detected in Baoding. Umc1545 and umc1125 were distributed on chromosome 7 (bin7.00 and bin7.04) and umc1061 were mapped on chromosome 10 (bin10.06). All the 3 loci could explain 24.20% of the phenotypic variation in total, and explain 8.91%, 7.47% and 7.82% of it, respectively. Compared with loci in Baoding, only 1 association locus phi299852 was detected distributed on chromosome 6 (bin6.07) and explained 7.22% of the phenotypic variation. For GCA of PH, 3 association loci umc1366, umc1492 and umc2342 were mapped on chromosome 9 (bin9.05, bin9.04 and bin9.05) and explained 9.29%, 9.14% and 9.14% of the phenotypic variation, respectively.

**Table 6:** Marker loci associated with GCA of GY, PH, EH.

Trait	Environment	Locus	Bin	Genetic distance	-log_10_(P)	R^2^(%)
Yield GCA	Baoding	umc1545	7.00	5.50	2.94	8.91
		umc1125	7.04	522.83	2.55	7.47
		umc1061	10.06	392.52	2.65	7.82
	Handan	phi299852	6.07	450.70	2.65	7.22
PH GCA	Baoding	umc1366	9.05	489.90	3.09	9.29
		umc1492	9.04	308.00	3.05	9.14
		umc2342	9.05	384.90	3.05	9.14

## Discussion

GY, PH and EH are complex quantitative characteristics. As an effective method, association analysis would accelerate the process for germplasm improvement and research of complex quantitative characteristics. Population structure, kinship, genetic diversity and LD could all influence the accuracy of association analysis [36].

### Population structure and kinship analysis

Xie et al. [37] showed that the structure of population would influence the extent of LD and association analysis and divided Chinese maize germplasm into 6 groups. In current study, research population of 120 inbred lines combined with a precise K was divided into 5 subpopulations (Table 4, Fig 1), among which Reid had the maximum proportion with 30 materials accounting for 25% of the materials and PB group was the second major population with 22 materials. All of the 5 groups contained core germplasm resources of China, indicating suitable materials for association analysis. Complex relative kinship could increase the level of LD and spurious association position. In current study, 58% inbred lines of experiment population had relative kinship value of 0, meaning that more than half of the lines had no kinship. This would decrease the false positive rate.

### Genetic diversity and PIC analysis

Genetic diversity was an essential factor for improving germplasm and studying complex quantitative characters [38]. In this study, all of the 120 inbred lines had 1,478 allelic gene variations and an average of genetic diversity of 0.7016 and PIC of 0.6593. Hao et al. [39] resulted an average of 0.69 of the PIC for 71 loci founded in Chinese modern wheat. Previous researches had been showed size of sliding window was consider as one factor affected the level of genetic diversity. It meant a large sliding window leaded high genetic diversity [40]. Higher genetic diversity and PIC ensured association analysis. The result of Xie et al. [37] showed that PIC value was correlated with LD level, which meant a higher LD level a locus (PIC>0.7) had compared with the locus (PIC<0.5).

### Linkage disequilibrium analysis

LD is the base of association analysis. A successful association analysis relies on the possibility of examining LD between the marker and the phenotypic traits of alleles associated with the maker [41]. In our research, all pairwise markers in experiment had an average of r^**2**^ and D’ of 0.041 and 0.480 respectively. Wang et al. [42] studied 145 SSR markers, and the result showed that 63.89% of the markers had an LD range of 18.75%-40.28%. Above all, the population in research had a feasible LD level.

### Association loci for yield, PH, EH and the combining ability

GY, as one of the most important emphases of maize, has made a gigantic breakthrough in terms of the use of heterosis. As a vital parameter for phenotype traits, the combining ability is taken more and more seriously by breeders. Xiang et al. [43] found GCA was more important than SCA, resulting that selecting for GCA in rice might be more effective. What’s more, the construction of QTL analysis and association mapping provided powerful measures for genetic improvement of grains. Plentiful loci of agronomic traits mapped on chromosomes offered an important theoretical foundation for marker—assisted selection (MAS) breeding. In this experiment, association analysis was used to detect GY-, PH-, EH- and GCA-related gene loci. A total of 20 association loci were identified and 7 association loci for GCA were detected with no locus detected for GCA of EH. The genetic diversity and PIC of the other loci were over 0.5 except umc1794 (bin9.05), and the Phenotypic contribution rate of the these loci were between 7.31%-9.29% which meant all of the these loci were identified as minor genes.

The 20 loci were compared with those of previous studies. Cai et al [44] uesd a set of 218 recombinant inbred lines (RILs) was used to evaluate PH, EH, PH/EH ratio and GY and grain yield components. In this study, there was one locus umc1710 (bin7.04) which was consistent with the results of Cai et al’s result. What’s more, according to the study, only one association locus mmc0241 (bin6.05) associated with GY was detected in both Baoding and Handan which belong to Zheng58 test population indicating that this locus could be stable expression and might avoid influence from the environment whereas no association site was detected in Chang7-2 cross population. The reason might be site mmc0241 was covered by the genetic background of Chang7-2. Combining with genotype of inbred lines and phenotype of F_**1**_ to detect the elite allele sites unacted on hybrid genetic background was a valuable orientation to research which might uncover the sites gathered from inbred lines whereas did not express owing to the masking of alleles interaction. Similarly, Wang et al. [45] detected one *qGY6b* locus mapped on bin6.05 and at the same time, heterotic locus *hlGY6b* was also mapped on bin6.05 using CSSL population. umc1061 (bin10.06) detected for yield in Baoding was also identified in GCA of yield which suggested that this locus might be without interference of genetic background and could be expressed stably in both inbred lines and F_**1**_. Locus umc0284 (bin5.05) was similarly associated with GY in Zheng58 test group in Baoding. Ding et al. [46] showed a QTL linked with test weight in bin5.05 using F_**2:3**_ population, and in this region, Silverio et al. [47] detected one QTL linked with endosperm hardness using F_**2**_ population. Starch was one essential component to yield and one QTL near starch biosynthetic genes was mapped in bin9.03 [48]. In this study, bnlg430 associated with GY in Handan was also mapped in bin9.03. This region might be a concentrated area for yield. However, no product was found to be related to the locus in maize GDB. Above all, these research results would provide momentous references for breeding programs applying the combining ability.

## Conclusions

In this study, the F_1_ population consisting of 240 hybrids was used for association mapping and 20 association loci for grain yield (GY), plant height (PH), ear height (EH) and GCA for the three traits were detected. These research results would provide momentous references for high yield breeding of maize and applying the combining ability.
